# The Digital Revolution in the Bakery Sector: Innovations, Challenges, and Opportunities from Industry 4.0

**DOI:** 10.3390/foods14030526

**Published:** 2025-02-06

**Authors:** Tsega Y. Melesse, Pier Francesco Orrù

**Affiliations:** Department of Mechanical, Chemical and Material Engineering, University of Cagliari, Via Marengo 2, 09134 Cagliari, Italy

**Keywords:** digitalization, bakery industry, automation, technological innovations, Industry 4.0

## Abstract

Industry 4.0 and digitalization are driving a major transformation in the bakery sector. This systematic review examines the latest advancements in digital technologies and platforms within the bakery industry. Innovations such as robotics, automation, blockchain, and wireless sensor networks are currently revolutionizing bakery operations by enhancing production efficiency, enabling real-time monitoring, and ensuring product traceability. Additionally, digital platforms are improving customer interactions through e-commerce, personalized product offerings, and targeted marketing strategies. Digitalization is also contributing to waste reduction, quality control improvement, and data-driven decision-making, leading to optimized inventory management and more efficient production automation. These advancements are fostering stronger customer engagement, resulting in cost savings and increased profitability. However, the sector faces several challenges, including resistance from companies to adopt new technologies, high implementation costs, a shortage of expertise, and concerns about preserving artisanal quality. This review provides valuable insights for researchers, businesses, and industry experts to deepen their understanding of how digitalization is shaping the future of the bakery sector while highlighting emerging opportunities, challenges, and avenues for future research.

## 1. Introduction

As a vital segment of the global food sector, the bakery industry has traditionally relied on artisanship, manual processes, and traditional techniques. However, the advancement in digital technologies and platforms has brought changes, driving efficiency, enhancing customer experiences, and addressing evolving market demands. In particular, digitalization improves productivity, lowers costs, and improves the customer experience. It transforms agri-food processing by increasing efficiency, product quality, sustainability, waste reduction, timely delivery to end users [1,2,3,4], as well as information dissemination [5]. Digital and web-based technological platforms in the agri-food supply chain include the Internet of Things (IoT), cloud computing, blockchain, digital twin, big data analytics, and sensor technologies [2,4,6]. Nowadays, the term “Automate or die” [7] is used to describe the rapid adoption of automation technologies in various industries, including agriculture and food. Despite being late adopters and lacking trained manpower, the agri-food industry is making progress toward automation [8]. Moreover, the transition to digitalization faces several challenges, including high initial investment costs, and integrating digital technologies within a traditional industry requires careful balance to maintain artisanal quality [9].

As the main contributor to the agri-food sector transitions to Industry 4.0, the bakery industry must adopt advanced digital solutions that can enhance automation and supply chain optimization processes to maintain competitiveness [10]. A survey conducted in 2022 revealed that approximately 46% of bakery industry participants are inclined to invest in new machinery and innovative products, while around 32% are considering investments in automation [11]. These advancements in bakery could increase efficiency across the business, improve data insights, facilitate better navigation of supply chain issues, reduce the risk of human error, and help meet evolving customer expectations [12]. Additionally, digital transformation is reshaping sales behaviors, prompting bakeries to invest in online platforms and e-commerce to expand their customer bases [13].

Digitalization could incorporate digital technologies like artificial intelligence (AI), big data, smart sensors, IoT, blockchain, robotics, digital twins, and virtual and augmented reality to mitigate the impact of the global health pandemic and environmental crises on food systems [14]. In the bakery industry, digitalization has been leveraged to enhance product quality, reduce waste, and automate processes. Furthermore, digitalization in bakeries is enabling remote support, automated material restocking, predictive maintenance, and quality control [15]. Smart technologies enable bakeries to control and monitor production in real time, demand forecasting, and direct interaction with customers through digital platforms [16,17,18]. Digitalization could also enable enhanced traceability and transparency in the supply chain, helping bakeries adhere to food safety regulations and solve issues related to consumer concerns related to sustainability [16,19].

Digitalization efforts in the agri-food sector are used to monitor the environment, crops, farming conditions, processing operations, and products throughout the entire supply chain [3,4,20,21], and very few publications have reported the role of digitalization in the bakery industry. Thus, this review aimed to address the following key research questions (RQs): What are the key digital innovations in the bakery industry (RQ1)? What challenges hinder bakery digitalization, and how can they be overcome (RQ2)? And what are the future perspectives on digital transformation in bakeries (RQ3)?

Accordingly, using a systematic approach, this review explored the cutting-edge developments and future possibilities of digitalization in the bakery sector, analyzing existing technological progress, recognizing innovations, and evaluating implementation challenges and future outlooks. The remaining part of the paper is structured as follows: Section 2 presents a review of the methodology, Section 3 discusses the results, and Section 4 summarizes the findings and implications.

## 2. Methodology

This section outlines the research methodology employed in this study, detailing the systematic approach used to identify, select, and analyze relevant literature. A structured search and selection process was implemented to ensure the inclusion of high-quality, peer-reviewed publications that contribute to the understanding of digital transformation in the bakery industry. Hence, Section 2.1 describes the research design and search strategy, including the databases, keywords, and filtering criteria used to retrieve relevant studies. Section 2.2 presents the selection criteria applied to refine the documents, ensuring that only studies directly relevant to bakery digitalization were included. Finally, Section 2.3 provides a descriptive analysis of the selected literature, highlighting key research trends, thematic distributions, and emerging areas of interest.

### 2.1. Design and Search Strategy

**Figure 1:**
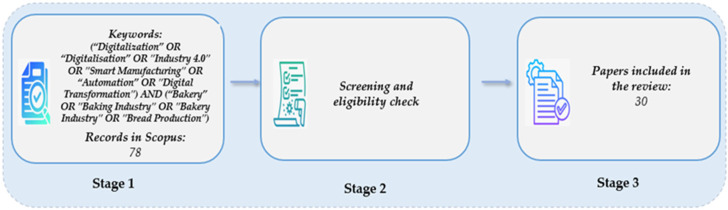
Key steps in the literature review process.

The search was conducted on Scopus using the article title, abstract, and keywords to identify peer-reviewed papers. Search terms used TITLE-ABS-KEY (“Digitalization” OR “Digitalisation” OR “Industry 4.0” OR “Smart Manufacturing” OR “Automation” OR “Digital Transformation”) AND (“Bakery” OR “Baking Industry” OR “Bakery Industry” OR “Bread Production”). Documents published between 2011 and November 2024 were identified following the methodological steps outlined in Figure 1. When it comes to document searches, the year 2011 marks the beginning of industrial digitalization, leading to process transformations and the rise of Industry 4.0 [22,23].

The systematic literature review process was used from initial keyword-based publications and searches to the final screening of papers. Initially, 78 records were retrieved, representing the total initial data. The second stage involves screening and eligibility checks to filter out irrelevant, duplicate, or low-quality papers based on specific criteria indicated below:

- Source Type: Peer-reviewed articles, book chapters, review articles, and conference papers are prioritized for academic credibility, comprehensive insights, and relevance to ongoing research in digitalization and bakery industries.
- Document Availability: Checks whether the full text of the document is accessible for reading. Only documents that are fully accessible and provide detailed information are included in the review. Documents with only abstracts, summaries, or restricted sources are excluded because they do not provide sufficient data for a detailed analysis of the topic.
- Relevance for digitalization in the bakery industry: This criterion evaluates whether the document addresses digitization aspects that are relevant for the bakery industry and focuses on automation, Industry 4.0 technologies, digital innovations, and digitization of the supply chain. Only sources that are directly related to the intersection of digital technology and baked goods or supply chain processes are included to align with the goals of the study.

### 2.2. Selection Criteria

Figure 1 illustrates the steps undertaken in the literature review process, structured into three main stages. In Stage 1, a set of keywords related to digitalization, Industry 4.0, smart manufacturing, automation, and digital transformation in the bakery sector was used to retrieve relevant records from the Scopus database, identifying 78 potentially relevant studies. Stage 2 involved a screening and eligibility check, in which the retrieved papers were assessed based on predefined criteria. This process included reviewing abstracts, methodologies, and findings to exclude studies that did not meet the inclusion requirements. Finally, in Stage 3, 30 papers were selected for the final review, forming the foundation of the study and offering valuable insights into the digital transformation of the bakery industry.

### 2.3. Descriptive Analysis

**Figure 2:**
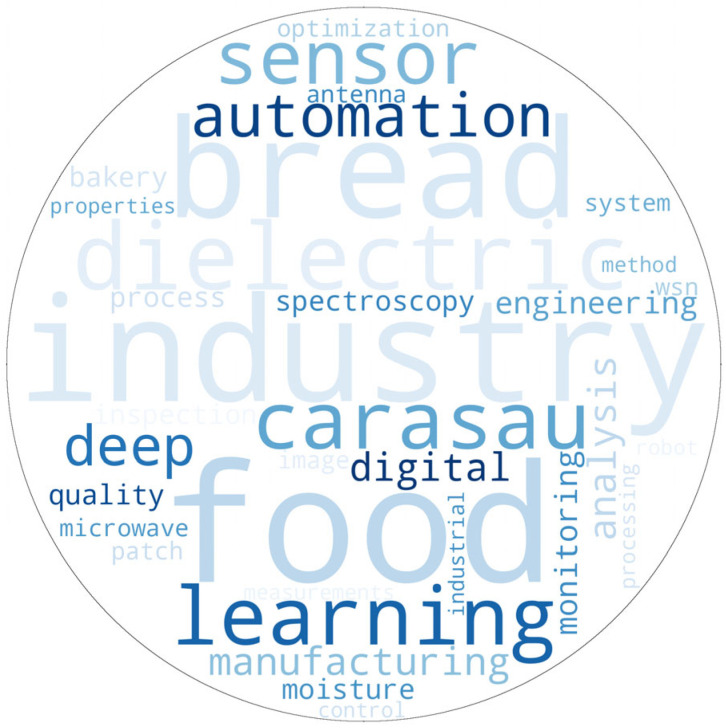
Most popular keywords in selected papers.

Figure 2 shows a word cloud illustrating recurring themes and keywords extracted from research papers focused on the bakery industry, food processing, automation, Industry 4.0, and related technologies. This visualization highlights the importance of certain topics in academic discourse. A notable keyword that stands out is “Carasau”, which refers to the well-known Sardinian crispbread [24,25].

The frequent occurrence of this term highlights the great research interest in automating the production of pane Carasau—a traditional bread that has cultural and culinary significance. Also known as “Carta da Musica” due to its thin, paper-like texture, Pane Carasau is a round, crusty bread with a remarkable shelf life, lasting up to six months [24,25]. This longevity, combined with its traditional roots, makes it a focus for studies aimed at reconciling automation with traditional food production.

Quality assurance and monitoring are becoming increasingly popular in digitalization research in the bakery industry, using terms such as microwave, dielectric, monitoring, inspection, quality, etc., indicating the growing research motivation in this area. Additionally, new tools are shown in the word cloud, including IoT and Wireless Sensor Networks (WSNs), Machine Learning (ML), Digital, Image, Model, Discovery, Deployment, etc., indicating the major efforts toward leveraging AI in this sector.

**Figure 3:**
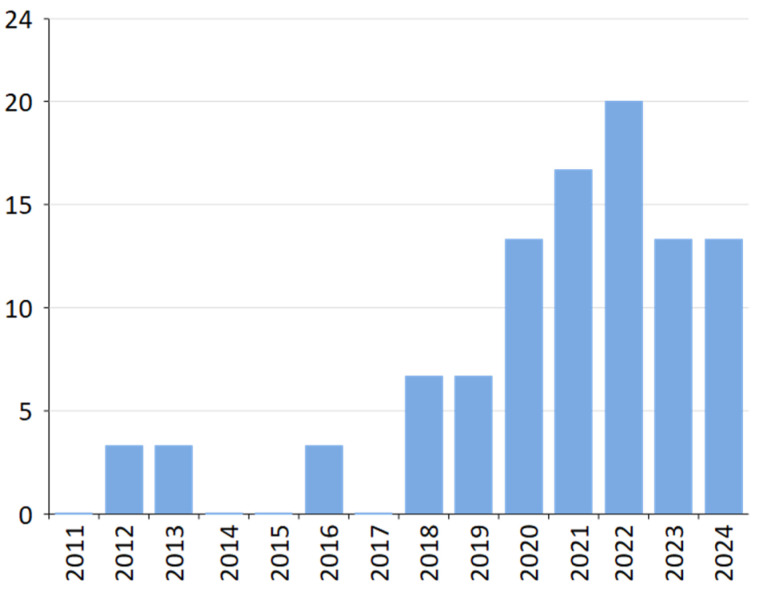
Annual scientific production.

As illustrated in Figure 3, the publication trend over the years reflects different phases of research activity. From 2011 to 2017, the number of publications remained low, indicating a foundational phase. In 2018, the number began to rise, indicating increased research activity. The momentum continued into 2019, with a steady increase.

The most significant surge occurred between 2020 and 2022, reaching its peak in 2022, when the number of publications remained consistently high, indicating a stable and mature phase of academic productivity. The number of publications shows a slight decrease after 2022. Accordingly, despite promising efforts from the scientific community, research into the digitalization of the bakery industry remains very limited.

**Figure 4:**
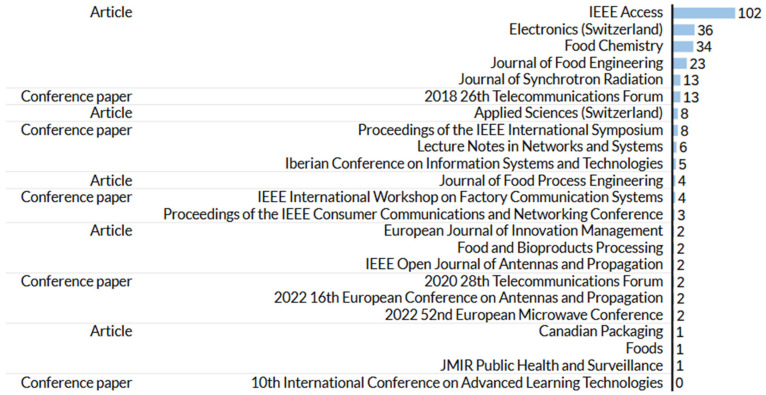
Citations per document type and source title.

Figure 4 shows the distribution of citation numbers in various academic and research sources and illuminates the awareness and effects of these publications. In particular, IEEE Access is the most influential source and collects 102 citations. This high number of citations underlines its significant role in the spread of research to a wide and diverse audience and stores several areas of study. Other leading scientific sources are *electronics (Switzerland), food chemistry, and the Journal of Food Engineering*, each of which has also shown significant influence in their respective areas. These sources underline the multidisciplinary character of research and reflect articles from engineering, chemical, and food science disciplines. The citation trends offer valuable insights into the relevance and reach of the published studies within the academic community and beyond.

**Figure 5:**
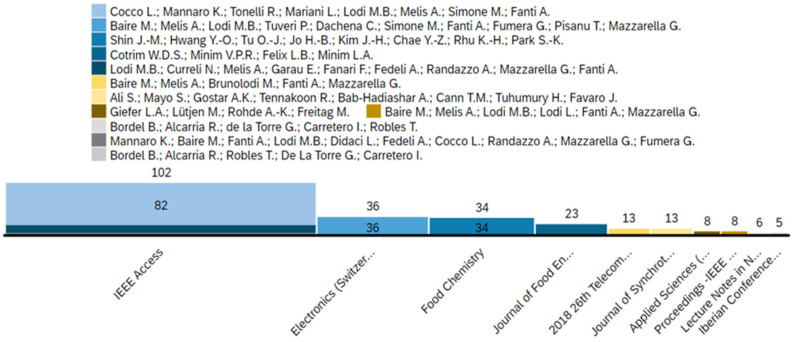
The distribution of publications across journals and conferences, with each colour indicating a specific group of authors.

Figure 5 illustrates the data distributions in a variety of academic sources and highlights collaboration in the research community. The visualization highlights the diverse range of partnerships between researchers and shows a robust and interconnected network spanning multiple disciplines. This interconnectedness not only reflects the collaborative nature of the field but also signifies the growing emphasis on multidisciplinary approaches in addressing complex research challenges in bakery digitalization. In addition, the diversity of journals shown in the graphic underlines the interdisciplinary nature of the work carried out, indicating research attention to a wide range of academic and professional areas, which indicates high attention in the field.

## 3. Discussion of Results

### 3.1. Technological Innovations in the Bakery Industry (RQ1)

**Figure 6:**
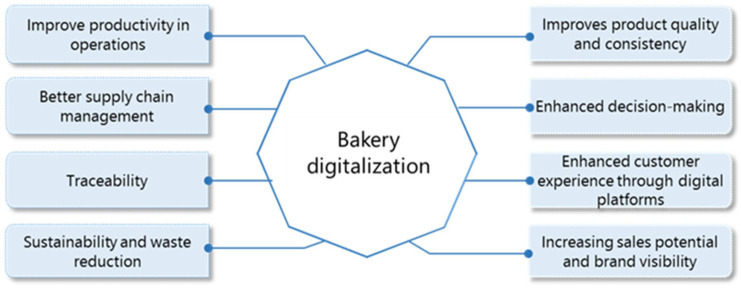
The main benefits of digitalization in the bakery industry.

Digitization in the bakery industry offers numerous advantages, including improved productivity, improved supply chain management, traceability, and sustainability efforts, as shown in Figure 6. It rationalizes processes, automates routine tasks, and enables real-time tracking to suppliers to ensure punctual delivery of raw materials and waste reduction. Digital tools also improve traceability and enable bakeries to monitor every step of the production process, ensure compliance with regulations, and build up consumers’ trust. Digital platforms also improve product quality and enable data-controlled decision-making. The customer experience is improved by personalized offers, efficient service, and optimized order systems.

Table 1 summarizes selected items concerning research focus and application purposes in the bakery industry. It provides an overview of bakery-related research, covering topics such as commercial bakery operations, value chain, small and medium enterprises (SMEs), bakery cashier’s point-of-sales, bread supply chain, dough formulations, baking stage, ergonomics, automation, logistics, family firms, Carasau bread production, pilot-scale electric oven research, bakery retail, etc. The key research areas include commercial bakery operations, dry-cooling processes, value chains, digital tools, bread supply chains, dough formulations, fermentation, and leavening monitoring, which improves product quality and reduces waste.

**Figure 7:**
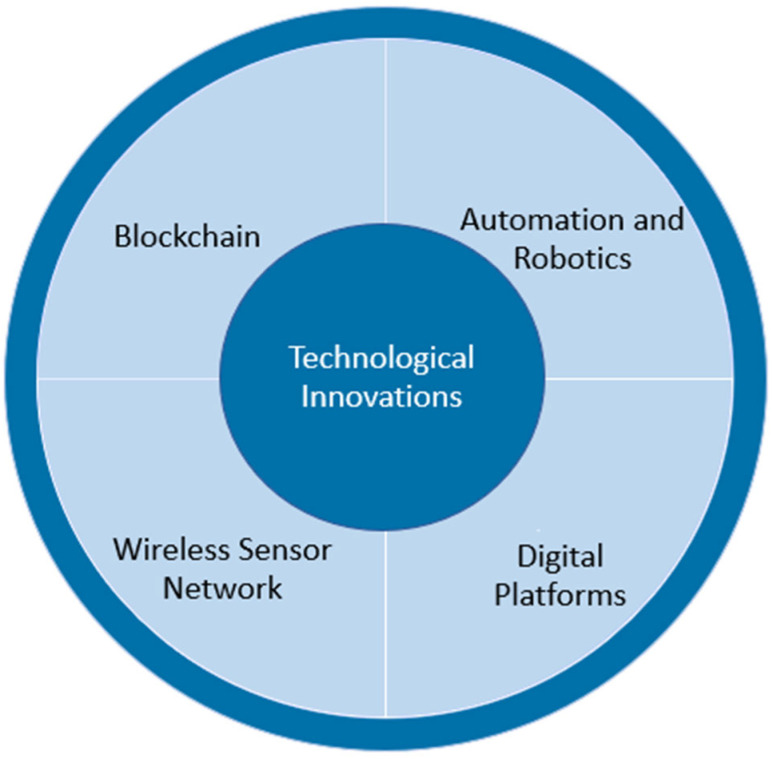
Key technological innovations identified in the bakery industry.

Automation and robotics, blockchain, WSN, and other digital platforms could improve the baking industry’s operational efficiency, reduce waste, and promote sustainability. This section discusses the latest developments in bakery digitalization, with a focus on the tools of digital technologies and innovations (Figure 7), as well as potential limitations and trade-offs of adopting such technologies.

#### 3.1.1. Automation and Robotics

Automation and robotics are enhancing operations in the bakery sector by improving efficiency, reducing manual labor, and ensuring high product quality [41]. According to the Food Engineering’s State of Food Manufacturing Survey, automation can increase throughput by 10–15% [8]. Specifically, studies show potential improvements in bakery operations like dough mixing, fermentation, and baking processes [35,39,47,49]. Moreover, automation in bakery processing lines offers several benefits, including higher output, worker health and safety, quality control, improved food safety, reduced labor costs, as well as traceability and compliance [8,27] through ergonomic design, wearable device monitoring, virtual reality training, and data-driven optimization [27]. Robots are being used in bakeries to perform many tasks, giving relief to human workers for more complex roles [30]. Despite challenges like adapting to process variability and computational intensity, robotics is increasingly being adopted across various food industry sectors, including bakeries, to address labor demands and improve efficiency [41]. Currently, automation in bakery sales is a key development to reduce worker demands and prevent disease spread [31,41]. Faster R-CNN-based point-of-sale modules are also used in bakeries to expedite order encryption and improve retail productivity [31]. Similarly, convolutional neural networks (CNNs) are being used to monitor bread transformations during baking, allowing for a better understanding of texture and color changes due to moisture loss and Maillard and caramelization reactions [36]. In addition, an ML approach has been implemented to automate data segmentation and analysis, enabling faster experiments and a better understanding of porous materials like bread doughs [39]. Process automation using the YOLOv5 algorithm is another development in the field to evaluate bread quality by combining shape and volume metrics with deep learning techniques [9].

Automation efforts extend to traditional products like Carasau bread. Studies using microwave spectroscopy for assessing dough properties show its potential to improve product quality while reducing wastage [47]. The study reveals variations in permittivity linked to dough composition, forming a third-order Cole–Cole model for leavening monitoring, and related work has highlighted the role of dielectric spectroscopy in the production automation of bread dough production [45]. Similar efforts have shown an automated dough fermentation monitoring system, utilizing ML and super ellipsoid model fitting and a movable laser sensor to estimate dough volume and track fermentation progress [43]. Studies have also shown the power of automation to improve production and value chain [16], which has been shown to enhance efficiency and user satisfaction.

The food industry integrates automation and robotics to improve productivity [50]. However, it presents challenges such as considerable capital investments, lack of flexibility for manual and tailor-made products, dependence on qualified workers, and energy consumption [51]. Automation can reduce labor costs but also create demand for qualified technicians. Companies must carefully consider production volume, market demand, and financing options before committing to automation, as the initial investment may take years to yield a return on investment [52]. Moreover, the transition of human employees to robot roles and the adaptability of robots to the variability of tasks is still a challenge [53].

#### 3.1.2. Blockchain

Blockchain is an electronic diary containing all the information and transactions related to the product or the process in chronological sequence [54]. It can improve productivity in the bakery industry by ensuring traceability, increasing operational performance, and building trust between customers and stakeholders. More specifically, blockchain could transform bakeries through improved transparency, efficiency, and trust by providing immutable and integrity-protected data without the need for third-party intermediaries [19]. One of the key applications of blockchain is tracking the journey of raw materials, ensuring that customers can verify the origin and quality of their products. It can also enable consumers to access detailed product histories, thereby enhancing trust and brand loyalty. Nowadays, it is not also new to see that blockchain-enabled payment systems further streamline transactions by reducing processing fees and delays, thus empowering bakeries to optimize operations and strengthen relationships with consumers and stakeholders.

Studies demonstrate the role of blockchain in improving efficiency within the bakery industry. For instance, a blockchain-based system has been proposed for managing the Carasau bread supply chain, aiming to achieve transparency and traceability [19]. Such studies have underscored its ability to address data quality gaps using technologies like radio frequency identification (RFID), smartphones, and IoT applications. Inline, two simple designs of Near Field Communication (NFC) and RFID devices have been proposed to implement blockchain technology to improve traceability during bread production processes [19,31].

Public blockchains like Ethereum and Bitcoin face slow transaction speeds and high latency due to proof of work (PoW) [55], which hinders real-time bakery supply chain processing. Private or consortium blockchains, such as Hyperledger Fabric, enhance performance but reduce decentralization [56]. PoW also increases energy consumption and operational costs [57], while private blockchains lower energy use but may pose security risks [19]. Bakery management systems (e.g., ERP, IoT) may lack direct blockchain compatibility [58], requiring middleware that adds complexity and costs [59]. Blockchain’s immutability complicates the General Data Protection Regulation, and food safety compliance [60] is another challenge that makes data modification difficult. Off-chain storage can mitigate this issue but reduces transparency [58].

#### 3.1.3. Wireless Sensor Networks

Industry 4.0 is transforming traditional bread manufacturing by leveraging WSNs and the IoT to enhance business value. WSNs enable real-time monitoring, predictive maintenance, and process optimization in the bakery industry [44]. Moreover, WSNs and related tools have shown significant potential to enhance monitoring, control, and optimization in the bakery industry. Research has demonstrated the efficacy of WSNs in monitoring bread-baking processes [17,27,28]. For instance, a pilot-scale electric oven integrated with a WSN has been developed to enhance baked food quality assessment [38]. This system utilizes digital images and filler temperature data to control oven temperature and belt velocity, ensuring consistent quality. Similarly, studies on Carasau bread production have reported the successful application of WSNs for real-time data collection, cost-effective electronics, and user-friendly interfaces aimed at process optimization in small-scale bakeries [34,35,44,45,47]. Furthermore, automatic image segmentation methods have been applied to the same product, ensuring quality control through efficient ML algorithms and image acquisition systems. These methods have achieved accurate segmentation and estimation [32]. A study has reported an ML-based application that employs object detection models to identify, classify, and count baked goods [5], thus enabling bakers to monitor unsold goods, optimize production, and enhance resource efficiency. Additionally, an online monitoring system utilizing a model-based PID controller has been introduced for the proofing process in bread baking [29], which can measure dough volume, correct for size variations due to yeast addition, and reduce the need for human intervention, thereby improving productivity and product quality. Through such data acquisition tools, promising results have been reported by [61] on optimizing bread-making processes based on crust color acceptability, enabling bread to be produced with crust colors of maximum acceptability.

WSNs in bakeries monitor environmental conditions, but battery-powered sensors need frequent replacement [59], while energy-harvesting alternatives are less reliable [62]. Wireless protocols like Wi-Fi, Zigbee, and LoRaWAN face interference from bakery equipment [62], whereas 5G or wired options enhance reliability but raise costs. There are also cases related to sensor drift, which is a low-frequency change in a sensor over time that can affect accuracy, potentially compromising food quality [62]. While high-precision sensors improve performance, they also increase costs. Wireless networks are susceptible to cyber threats [63], and although encryption and blockchain enhance security, they introduce additional computational overhead [60].

#### 3.1.4. Digital Platforms

Digital platforms in the food industry are increasingly connecting stakeholders, streamlining operations, and enhancing customer experiences, all while improving supply chain efficiency. These platforms facilitate various aspects such as online food delivery, inventory management, and restaurant reservations. Moreover, digital tools have the potential to speed up the time to market for new food products [64].

Bakeries must adapt by moving traditional offerings to digital tools, promoting products, and leveraging social networks for customer engagement [13]. The rise of digital technologies in the bakery industry has significantly transformed work, communication, and consumption in the food industry, particularly with the introduction of online food delivery services. The DIGIFOOD dashboard, for instance, monitors local and online bread and pastry and allows for comparisons between local and online food outlets [18]. It can also highlight high-priority areas with limited access, facilitating targeted improvements. A case study of “ArteBianca Delivery”, a family bakery firm in South Italy, demonstrates how digital tools have transformed marketing, delivery, e-commerce, and customer care, offering practical insights for other companies implementing digital strategies [17]. There is also a case study in which the catering and retail industry is leveraging digital platforms to enhance value and upgrade its operations [33]. A related study in Hungary used digital platforms in bakeries for the Food Choice Questionnaire and customer interaction [46]. Similarly, digitalization improves Life Cycle Assessment (LCA) by allowing machine-readable Environmental Product Declarations and automating data handling, which supports statistical analyses, benchmarking, and error identification for effective and scalable uses [5].

While there are advantages to using digital platforms for bakery digitalization, there are also drawbacks. The initial cost of implementation—including software, hardware, and training—can be high, and ongoing maintenance and updates add to long-term expenses, which may be difficult for smaller bakeries to justify. Integrating digital systems with existing equipment can be complex, and employees need training, which may disrupt operations and face resistance [65]. Data security is another concern, with increased risks of breaches and the need to comply with regulations, which could require additional resources [66].

Scalability and platform flexibility are also important, as some systems may become restrictive as the bakery grows, and customization options can add complexity to maintenance. While automation improves efficiency, it could reduce personal interactions that many customers value, potentially impacting loyalty [67]. Lastly, the environmental impact of digitalization, such as energy consumption from cloud services, should be considered alongside the operational benefits [68].

### 3.2. Challenges and Strategies (RQ2)

The baking sector faces challenges in digital transformation due to resistance from companies, high implementation costs, lack of trained staff, integration difficulties, and market saturation, as well as ergonomic problems and the integration of various technologies into traditional setups [69,70,71]. Moreover, as defining a transformation map is becoming a great challenge for many companies [72], bakeries should start creating clear roadmaps toward digitalization. Traditional bakeries often resist the introduction of digital tools since there is a lack of expertise or concerns about the loss of their artisan quality. According to studies, consumers still prefer traditional bakery bread compared with packaged baked goods made by commercial bakeries [13]. Moreover, implementing digital solutions can be prohibitively costly for small-scale bakeries. Addressing issues related to digital literacy and technology development challenges, such as internet access, especially in rural areas, is crucial for the equitable application of these tools [73]. Similarly, real-time data acquisition in the food industry is hindered by the biological complexity of food items, sensor restrictions, data integration problems, high initial investment, and regulatory requirements [74]. Overcoming this requires new developments in sensor technology, better data integration, and stronger cooperation between stakeholders.

The bakery industry faces distinct challenges depending on the scale of operation, as illustrated in Table 2. Artisan bakeries often struggle with supply chain management due to manual inventory control and limited supplier networks, whereas large-scale producers benefit from advanced systems but contend with significant operational costs [75,76]. Affordable inventory tools and collaborative supplier networks can support artisans, just as predictive analytics and blockchain can enhance industrial efficiency [77]. For digital adoption, financial and expertise constraints hinder artisans, whereas large-scale producers require substantial investments to integrate new technologies [78]. Artisans can start with low-cost digital tools, and in contrast, industrial producers should implement comprehensive ERP systems. Addressing skill gaps, smaller bakeries need basic training to improve digital literacy, while major producers must hire specialized staff and prioritize continuous development. Collaborative training programs and professional development initiatives can bridge these gaps. Customer engagement also presents varying difficulties. Artisans often struggle to establish an online presence [17], yet large producers leverage big data analytics effectively. Social media and e-commerce platforms can help smaller bakeries connect with customers, whereas data-driven marketing enhances industrial outreach. Regulatory compliance is another challenge—artisans grapple with resource limitations, and in contrast, industrial producers navigate complex global standards. Cost-effective traceability tools like QR codes and advanced blockchain systems offer solutions [79]. When balancing personalization and adaptability, artisans emphasize a personal touch, whereas large-scale businesses focus on automation for scalability. Digital tools tailored to smaller operations and AI-driven systems for industrial producers can enhance customer satisfaction [80]. Lastly, cybersecurity remains an overlooked issue for artisans but a priority for large enterprises [81]. Basic security measures can safeguard small businesses, while industrial producers must invest in robust systems.

### 3.3. Future Outlook (RQ3)

Future opportunities include creating hybrid production models that combine traditional artisanal techniques with smart manufacturing to guarantee quality and efficiency. To maximize the efficiency of production planning, research-driven strategies should focus on reasonably priced digital solutions designed for small- and medium-sized bakeries, such as inexpensive IoT systems and AI-driven demand forecasting tools. Blockchain-enabled smart ingredient-tracking system advancements could also improve food safety and traceability while addressing regulatory concerns [77,80]. Moreover, collaborations between academic institutions, technology providers, and bakeries can promote knowledge sharing and accelerate the adoption of new technologies. Next research should also focus on energy-efficient baking technologies, waste-reducing ingredient formulations, and digital platforms that enable direct-to-consumer distribution channels, with sustainability serving as a major motivator. The additional transformation could be made possible by a well-organized policy framework that encourages digital adoption in the bakery industry and offers financial incentives to SMEs. Developing standardized digital transformation frameworks specific to bakeries could guide SMEs in adopting digital tools effectively.

## 4. Conclusions

Digitalization could enable bakeries to optimize production processes, enhance customer experiences, and meet evolving consumer demands. Innovations like automation, blockchain, WSNs, and emerging digital platforms have improved production and supply chain management, addressing food safety, traceability, and sustainability concerns. Digital tools in bakeries offer real-time monitoring, quality control, and traceability, potentially improving product quality, reducing costs, and ensuring transparency. Nevertheless, digital transformation faces challenges such as high initial investment costs, lack of trained personnel, limitations with sensor quality, food product complexity, and companies’ resistance to new technologies, particularly in small-scale and artisanal bakeries. Moreover, integrating digital solutions into traditional processes requires a careful balance to preserve artisanal values.

To overcome these issues, bakeries should adopt phased digitization strategies, beginning with cost-effective automation tools and progressively investing in advanced technologies to ensure a return on investment. Collaborating with digital solution providers can also help mitigate initial costs, while investing in employee training programs will facilitate the successful implementation of new technologies. Moreover, bakeries should prioritize consumer engagement through digital marketing platforms to foster brand loyalty and respond to shifting consumer preferences. In addition, keeping a balance between adopting new technologies and preserving traditional product quality will be crucial in maintaining the distinctive qualities of artisan products while driving innovation.

Looking to the future, the bakery industry will be shaped by continued advancements in automation, data analytics, and digital marketing. As consumer preferences shift toward sustainability and operational efficiency, bakeries must adapt by incorporating these new technologies while prioritizing consumer engagement. This work offers valuable insights for bakeries, emphasizing the need for gradual digital adoption, workforce development, and external collaboration to navigate the digital transformation. The sector should prioritize consumer engagement and brand loyalty through digital platforms while ensuring that new technologies complement rather than compromise traditional values. By adopting these strategies, bakeries can remain competitive, efficient, and responsive in an increasingly digital world. As the industry continues to evolve, further exploration into the sustainability of digital technologies in bakeries will be critical in ensuring long-term environmental and economic viability.

## Figures and Tables

**Table 1:** Summary of selected papers in terms of the research focus and application purposes.

Focus Area	Application Purpose	Ref.	Focus Area	Application Purpose	Ref.
Commercial bakery	Tracking unsold baked goods	[26]	Ergonomics, automation, and logistics	Improve the safety and productivity of workers	[27]
Drying-cooling process	Automation of Carasau bread production	[28]	Bakery	Real-time process control	[29]
Value chain	Improving efficiency through digital services	[16]	Supply chain	Digitalisation of LCA	[5]
Small and medium Enterprises	A robot used for the movement of dough	[30]	Family firms	Customer interactions, delivery, and marketing	[17]
Bakery cashier’s point-of-sales	Detection, classification, and receipt generation	[31]	Carasau bread production	Quality control	[32]
Bread supply chain	Bread quality evaluation	[9]	Bakery retail	Product identification and face recognition system	[33]
Production process	Process monitoring	[34]	Carasau bread manufacturing	Process monitoring	[35]
Baking stage	Classification of browning degree of bread	[36]	Traditional bakery	Traceability system	[19]
Bakery store	Automation, self-payment cashiers	[37]	Pilot-scale electric oven	Baked food evaluation	[38]
Dough formulations	Automatic segmentation of porous bread dough	[39]	Bakery	Production, management, and product delivery	[40]
Sale point	Bakery assistant robot	[41]	Bakery	Plant control	[42]
Dough Fermentation	Monitoring of the fermentation process	[43]	Carasau bread production	Improvement in the hardware and software module	[44]
Monitoring of leavening	Development of a microwave inline system	[45]	Breads and bakery products	Food Choice Questionnaire digitalization	[46]
Food outlets	Digitalization of local food environments	[18]	Bakery	Optimization of the bread-making process	[47]
Bread production	Assessment of aromatic compounds	[48]	Carasau bread	Automation of quality monitoring	[47]

**Table 2:** The summary of key challenges in small-scale artisan bakeries and large industrial producers.

Aspect	Artisan Bakeries	Large-Scale Producers	Major Differences
Supply chain	Manual inventory control and limited supplier networks [31,82]	Integration of advanced systems [83]	Artisans depend on informal ties; industrial producers leverage scalable digital systems
Digital system	Limited budgets for adopting advanced digital tools and systems [84]	High initial costs but strong financial capacity to invest [13]	Small bakeries face greater challenges with affordability and expertise than large producers
Skill gaps	Limited digital literacy among owners and staff [30,43,85]	Need for specialized staff to manage and maintain complex systems [8,86]	Artisans need basic training, while industrial producers need specialist knowledge
Customer interaction	Difficulty in establishing an online presence and digital marketing [33]	Opportunity to leverage big data analytics for customer insights [75]	Artisans prioritize local loyalty; industries navigate diverse markets
Regulatory compliance	Challenges in implementing digital traceability for compliance [87]	Pressure to meet global standards often drives major system upgrades [13]	Artisans face knowledge gaps; large producers tackle complex global regulations
Personalization and adaptability	Personalization is key, but resources for scalable digital tools are lacking [88]	Automation drives mass customization through data [69,89]	Artisans prioritize personal touch; industrial producers focus on flexibility at scale
Cybersecurity	Cybersecurity measures are not widely known or used [90]	Substantial investment in safeguarding sensitive data [19]	Artisans often neglect cybersecurity, while industrial producers see it as vital

## Data Availability

No new data were created or analyzed in this study. Data sharing is not applicable to this article.
